# Fragmentation of Pooled PCR Products for Highly Multiplexed TILLING

**DOI:** 10.1534/g3.119.400301

**Published:** 2019-06-18

**Authors:** Andrea Tramontano, Luka Jarc, Joanna Jankowicz-Cieslak, Bernhard J. Hofinger, Katarzyna Gajek, Miriam Szurman-Zubrzycka, Iwona Szarejko, Ivan Ingelbrecht, Bradley J. Till

**Affiliations:** * Plant Breeding and Genetics Laboratory, Joint FAO/IAEA Division of Nuclear Techniques in Food and Agriculture, IAEA Laboratories Seibersdorf, International Atomic Energy Agency, Vienna International Centre, PO Box 100, A-1400 Vienna, Austria and; † Department of Genetics, Faculty of Biology and Environmental Protection, University of Silesia, Jagiellonska 28, 40-032, Katowice, Poland

**Keywords:** TILLING by Sequencing, DNA shearing, sonication, *Hordeum vulgare*, barley

## Abstract

Improvements to massively parallel sequencing have allowed the routine recovery of natural and induced sequence variants. A broad range of biological disciplines have benefited from this, ranging from plant breeding to cancer research. The need for high sequence coverage to accurately recover single nucleotide variants and small insertions and deletions limits the applicability of whole genome approaches. This is especially true in organisms with a large genome size or for applications requiring the screening of thousands of individuals, such as the reverse-genetic technique known as TILLING. Using PCR to target and sequence chosen genomic regions provides an attractive alternative as the vast reduction in interrogated bases means that sample size can be dramatically increased through amplicon multiplexing and multi-dimensional sample pooling while maintaining suitable coverage for recovery of small mutations. Direct sequencing of PCR products is limited, however, due to limitations in read lengths of many next generation sequencers. In the present study we show the optimization and use of ultrasonication for the simultaneous fragmentation of multiplexed PCR amplicons for TILLING highly pooled samples. Sequencing performance was evaluated in a total of 32 pooled PCR products produced from 4096 chemically mutagenized *Hordeum vulgare* DNAs pooled in three dimensions. Evaluation of read coverage and base quality across amplicons suggests this approach is suitable for high-throughput TILLING and other applications employing highly pooled complex sampling schemes. Induced mutations previously identified in a traditional TILLING screen were recovered in this dataset further supporting the efficacy of the approach.

Next generation sequencing (NGS) techniques have had a profound impact on biological research. While technologies continue to advance, whole genome approaches remain costly for projects involving the analysis of species with large genomes or those involving the interrogation of many individuals. A variety of reduced representation genome sequencing approaches have been described to circumvent this issue (Hirsch *et al.* 2014). One powerful approach to evaluate sequence variation in targeted regions is by sequencing PCR amplicons. Amplicon sequencing has been applied for the discovery and characterization of both natural and induced mutations in plant and animal populations (Rigola *et al.* 2009; Tsai *et al.* 2011; Pan *et al.* 2015; Duitama *et al.* 2017). For example, sequencing has been used to increase throughput of mutation discovery for the reverse-genetics technique known as TILLING (Targeting Induced Local Lesions IN Genomes)(McCallum *et al.* 2000; Till *et al.* 2018). For TILLING, mutant populations are typically created using mutagens such as ethyl methanesulfonate (EMS) or a combination of sodium azide and N-Nitroso-N-methylurea (MNU) that induce primarily single nucleotide variants (SNV). While the frequency of mutations varies between species and ploidy level, mutation densities in diploid species have been reported to range between 1 mutation per 150,000 and 1 mutation per 700,000 base pairs (Jankowicz-Cieslak and Till 2015). TILLING screens typically aim to recover multiple mutations from a single target to increase the chances of recovering deleterious alleles. Therefore, a typical TILLING assay involves the screening of 3000 or more mutant individuals. To increase throughput, genomic DNAs are pooled together prior to mutation screening. The application of new sequencing technologies to improve TILLING throughput has been termed TILLING by Sequencing (Tsai *et al.* 2011). In addition to pooling genomic DNA, multiple amplicons are produced from each gDNA pool and combined prior to massively parallel sequencing to further improve throughput. The quantitative nature of NGS methods means that rare induced and natural SNV mutations can be effectively recovered from pools of over 200 individuals (Pan *et al.* 2015; Duitama *et al.* 2017). The most common sequencing platform used for TILLING by Sequencing is short read sequencing-by-synthesis from the company Illumina (Tsai *et al.* 2011; Guo *et al.* 2015; Pan *et al.* 2015). Similar approaches have been used in population genetics studies to identify rare alleles in large germplasm collections (Duitama *et al.* 2017).

Direct sequencing of PCR amplicons is potentially advantageous in that an even read coverage across the target can theoretically be achieved, thus providing consistent recovery of sequence variants. This approach is disadvantageous, however, due to the fact that the length of sequenced amplicons is limited to the maximum read lengths of the sequencer used (*e.g.*, 500-600 bp for the Illumina MiSeq)(Pan *et al.* 2015; Duitama *et al.* 2017). Thus, multiple amplicons are required to screen an entire gene, necessitating extra liquid handling steps and also additional work in adjusting PCR amplicon concentrations to a similar level prior to sequencing. An alternative approach is to produce longer PCR products and to fragment them prior to library construction. Several approaches for fragmentation have been described such as nebulization, enzymatic cleavage, and ultrasonication (Head *et al.* 2014). Most approaches are developed for the fragmentation of genomic DNA samples that contain high molecular weight molecules. We describe here the optimization of ultrasonication for PCR products amplified from the genomes of *Coffea arabica* (Arabica coffee) and *Hordeum vulgare* (barley) ranging between 670 and 1513 bp. We further show suitable coverage can be achieved in a pool of 32 distinct PCR products generated from PCR amplification of pools of 256 genomic DNA samples prepared from chemically mutagenized barley. This suggests that the approach can be easily adapted for pooled amplicon sequencing in different species.

## Materials and Methods

### Genomic DNA and PCR

**Figure 1:**
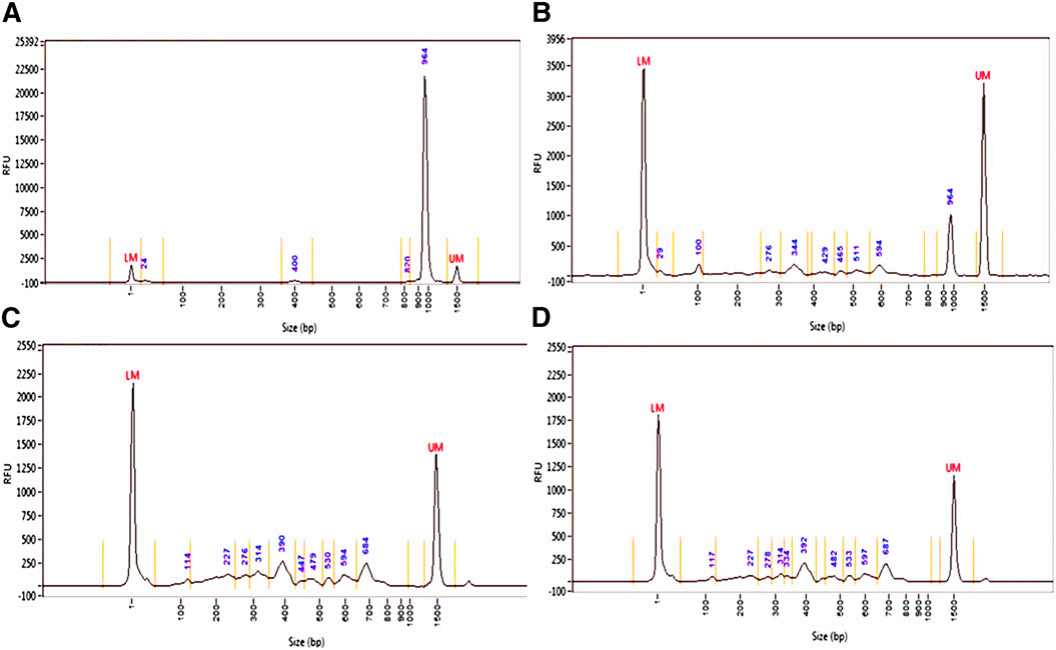
Comparison of different fragmentation parameters of a single PCR amplicon. Panel A shows the non-fragmented control of the single 964 bp PCR product as assayed by capillary electrophoresis. Panel B shows the results of test parameter 1 in Table 1. This is an example of incomplete fragmentation as the original 964 bp PCR product can be observed at a high concentration. Panel C and D are technical repeats showing results of fragmentation using test #13 parameters. The y axis in all graphs shows relative abundance of product in relative fluorescence units (RFU). The x axis shows molecular weight in base pairs. LM and UM are the lower and upper marker, respectively.

Genomic DNA from *Coffea arabica* was provided by Margit Laimer. PCR primers were designed using the Primer3 program with parameters previously developed for TILLING assays(Till *et al.* 2006b). Primer sequences were TCGATTCGATTCGTTGACACCCCTA and TGGATGATGGATGGGAATGTGGTTC. PCR was performed as previously described (Till *et al.* 2015). Amplification of a single PCR product was assayed using agarose gel electrophoresis and confirmed via capillary electrophoresis (Figure 1A).

Genomic DNA from chemically mutagenized barley was prepared using a modified CTAB protocol (Szarejko *et al.* 2017). DNA samples were adjusted to a similar concentration and pooled. Two hundred and fifty-six samples were combined in each pool. Forty-eight unique pools were prepared. Primers were designed to target regions of the *H*. *vulgare* genome as previously described (Table S1)(Slota *et al.* 2017). PCR primers amplify part of a coding sequence and thus target names are assigned for each amplicon that is unqiue from gene annotations. PCR was performed on pooled genomic DNA samples using 32 unique primer pairs. Individual PCR reactions were performed for each primer pair. The concentration of each PCR product was estimated by running products on a 96-well eGel system with lambda DNAs standard of known concentrations (Huynh *et al.* 2017). Average concentration was estimated visually and all samples adjusted to a final concentration of 200 ng in TE buffer. PCR products were then pooled together. DNA sequences of each resulting amplicon were prepared from the barley reference genome and combined into one multifasta file for downstream bioinformatic analysis (Gupta *et al.* 2017). GC content for each amplicon was extracted from the multifasta file using the Emboss infoseq tool (Carver and Bleasby 2003). Average GC content for barley was calculated from the coding sequences from the International Barley Genome Sequencing Consortium version 2 assembly (IBSC v2, INSDC Assembly database version 95.3) (International Barley Genome Sequencing Consortium *et al.* 2012).

GC percentage for each sequence was extracted using the Emboss infoseq tool. Minimum and maximum GC percentage was extracted using the following command: awk ′BEGIN{first = 1;} {if (first) {max = min = $3; first = 0; next;} if (max < $3) max=$3; if (min > $3) min=$3;} END {print min, max}′. Average GC was calculated using the following command: awk ′{sum = sum+$3 ; sumX2+=(($3)^2)} END { printf “Average: %f. Standard Deviation: %f \n”, sum/NR, sqrt(sumX2/(NR) - ((sum/NR)^2))}′ .

### Sonication of PCR products

Sonication of PCR products was performed using a Covaris M220 ultrasonicator with microTUBE AFA bead split tubes (coffee amplicons) or microTUBE AFA FiberPre-slit (barley amplicons) in 60 μl volumes except where indicated with parameters adjusted according to Tables 1, S2 and S3. Fragmentation of PCR products was assayed using a Fragment Analyzer with the low sensitivity 1kb separation matrix with 30 cm capillaries (cat #DNF935). Analysis of data were performed using the *PROSize* Data Analysis Software. Performance of sonication of pooled PCR products was independently tested in 48 pools. Pooling of genomic DNA for each of the 48 pools was done such that each individual genomic DNA is represented in triplicate in the experiment (in three different pools). A total of 4096 DNAs from unique mutant lines are represented in the 48 pools.

**Table 1:** Test parameters for sonication of a 964 bp PCR product

Test #	Time [s]	Peak power [W]	Duty Factor [%]	Cycles/burst	Volume [µl]	Average power [W]
1	30	75	20	50	60	15
2	30	75	20	350	60	15
3	30	75	20	750	60	15
4	30	75	5	200	60	3.75
5	30	75	25	200	60	18.75
6	30	75	12.5	200	60	9.38
7	30	20	50	200	60	10
8	30	30	50	200	60	15
9	30	40	50	200	60	20
10	15	75	20	200	60	15
11	45	75	20	200	60	15
12	60	75	20	200	60	15
13	30	50	40	200	60	20
14	30	75	5	1000	60	3.75

### Sequencing and data analysis

Quantified PCR products were normalized to approximately 200ng and pooled together according to a three-dimensional pooling scheme whereby samples were arrayed in a 16 × 16 × 16 grid for pooling (Gupta *et al.* 2017). A total of 48 pools were created for library preparation. Sequencing libraries were prepared using TruSeq Nano DNA HT Library Prep Kit (Illumina, Catalogue # FC-121-9010DOC) with 200 ng of starting pooled PCR products according to manufacturer’s recommendations. Libraries were quantified using a Qubit fluorimeter, concentrations normalized to a common molarity and pooled as previously described (Datta *et al.* 2018). Sequencing was performed on an Illumina MiSeq using 2x300 PE chemistry according to manufacturer’s protocol. Reads were mapped to amplicon sequences during the sequencing run using BWA with the MiSeq BWA default settings (Li and Durbin 2009). Sequencing coverage and mapping quality were calculated using Qualimap version v.2.2.1 with default settings except that the number of windows was set to amplicon length to retrieve per base metrics used in Figure 3 (Okonechnikov *et al.* 2016). Base qualities were prepared using pysamstats and the baseq feature (https://github.com/alimanfoo/pysamstats). Mean coverage data per amplicon for all 48 pools was extracted from the Qualimap output. Principle component analysis of all 48 pools was produced using the multi-sample BAM QC feature of Qualimap. Correlation coefficients were calculated using the LibreOffice CORREL function. Coverage data used to calculate mean coverage for tomato amplicon sequencing comes from Gupta *et al.* (2017). Coverage data used to calculate mean coverage for cassava sequencing was extracted using nucleotide number binning rather than amplicon name as data were mapped to a contiguous sequence rather than individual amplicons (Duitama *et al.* 2017). Data for barley, tomato and cassava were collected on the same MiSeq instrument using 2x300 PE sequencing.

Screening of data for previously identified SNP mutations was performed using the CAMBa pipeline for three dimensionally pooled samples as previously described (Tsai *et al.* 2011; Gupta *et al.* 2017). The HaplotypeCaller tool from GATK 4.0.10.0 was used as a second method to call SNPs (McKenna *et al.* 2010). Processing was carried out in parallel using the following command: ls *.bam | parallel java -jar gatk-package-4.0.10.0-local.jar -Xmx32g HaplotypeCaller -I {} -R reference.fa -O {.}.vcf–sample-ploidy N, where sample ploidy N was set to either 600 or 2000 in different analyses (Tange 2011). Resulting VCF files were first filtered to remove common variants appear above 5% allele frequency in pools (bcfftools view -i ‘AF < 0.05’). True mutations should be present in only one sample in a pool and therefore the expected frequency is 1/256 for homozygous mutations and 0.5/256 for heterozygous mutations. The 16 VCF files from a single dimension pool where then grouped together for further analysis. True induced mutations should appear only one time in each dimension. Text files containing variant positions were created, and variants unique to a single file were extracted using awk ′END {for (R in r) {split(r[R], t, SUBSEP) if (!t[1]) print t[3], t[2]}}{k = $1 SUBSEP $2 SUBSEP $3r[k] = c[k]++ SUBSEP FILENAME SUBSEP $0 }′ *.txt > output. This resulted in three files containing unique variant calls for each pooling dimension. True mutations should be present one time in each dimension and so should be represented in each of the three lists. Matching values fitting these criteria were extracted using grep -wFf. Original VCF files were evaluated in cases where positive control mutations were not identified using this method. This was how the mutation in VDE_2_27 in pool 14 was recovered.

### Data availability

The raw datasets analyzed during the current study are available in the Sequence Read Archive, Accession PRJNA422048 at https://www.ncbi.nlm.nih.gov/bioproject/422048. M_3_ seed from positive control mutants is available upon request while supplies last. Table S1 contains target names, primer sequences, amplicon lengths and percent GC of each amplicon. Table S2 contains pre-set parameters of sonication of genomic DNA. Table S3 contains additional tested sonication parameters. Table S4 contains sequencing statistics for mutant pool 10. Table S5 contains sequencing statistics for mutant pool 11. Table S6 contains sequencing statistics for mutant pool 15. Table S7 contains sequencing statistics for mutant pool 19. Table S8 contains sequencing statistics for mutant pool 20. Table S9 contains sequencing statistics for mutant pool 37. Table S10 contains sequencing statistics for mutant pool 41. Table S11 contains summary sequencing statistics for 48 barley pools. Table S12 contains coverage data and calculations for 32 barley amplicons in 48 pools. Table S13 contains coverage data from a tomato TILLING by Sequencing experiment using non-fragmented amplicons. Table S14 contains coverage data from cassava sequencing using non-fragmented amplicons. Table S15 contains frequencies of mutations called with the CAMBa and GATK HaplotypeCaller tools. Table S16 contains cost estimations for experiments using fragmented and non-fragmented pooled amplicons. Figure S1 shows Fragment Analyzer profiles of a single amplicon subjected to different default sonication settings. Figure S2 shows Fragment Analyzer profiles of a single amplicon subjected to different sonication parameters. Figure S3 shows Fragment Analyzer profiles of a single amplicon subjected to additional sonication parameters. Figure S4 shows Fragment Analyzer profiles of pooled amplicons before and after sonication. Figure S5 shows read coverage, base coverage and mapping quality for tomato amplicons not subjected to sonication. Figure S6 shows insert sizes for 48 barley sequencing pools. Figure S7 shows principle component analysis of sequencing statistics for 48 barley pools. Figure S8 contains a graph of mean coverage and amplicon size in 48 barley pools. Figure S9 contains a graph of mean coverage and percentage of GC in amplicons in 48 barley pools. Figure S10 contains a graph of percentage of GC and amplicon size in base pairs. Supplemental material available at FigShare: https://doi.org/10.25387/g3.8052821.

## Results

### Evaluation of sonication parameters established for genomic DNA on PCR products

A PCR product of 964 base pairs (bp) amplified from *Coffea arabica* genomic DNA was created for initial experiments to optimize DNA fragmentation. The aim of the work was to identify conditions resulting in a fragmented PCR product between 350 and 500 bp using a Covaris focused-ultrasonicator. The manufacturer’s parameters established for genomic DNA shearing were initially tested (Table S2). Fragment analysis showed limited fragmentation and retention of a high concentration of 964 bp product at all pre-defined parameters (Figure S1).

### Optimization of parameters for sonication of a single PCR amplicon

Based on the results from the default parameters for genomic DNA we hypothesized that halving the power, duty factor and/or cycles/burst may improve fragmentation of lower molecular weight PCR products. Fourteen distinct sonication parameter combinations were chosen and evaluated (Table 1). Test numbers 1 through 3 were designed to assess the effect of the number of cycles/burst on the fragment size and distribution. Tests 4 through 6 were designed to assess the effect of the duty factor on the fragment size and distribution. Tests 7 through 9 were designed to assess the effect of the peak power on the fragment size and distribution. Tests 10 through 12 were designed to assess the effect of the duration of the sonication on the fragment size and distribution, and tests 13 and 14 were designed to test the combination of high duty factor with an average number of cycles/burst and very low duty factor with the highest cycles/burst respectively.

The extent of PCR fragmentation was evaluated using a capillary Fragment Analyzer (FA), (Figure S2). Relative concentration of DNA fragments was evaluated owing to the fact that the absolute concentration varies according to amount of input PCR product used in the assay. Based on this analysis, parameters of test # 13 were chosen to provide the best fragmentation as they produced a broad distribution of fragments with a peak of 390 bp, which was close to 350 bp, one of two sizes recommended for Illumina library preparation (Figure 1). Additional modifications, whereby average power (Peak Power and Duty Factor) were held constant while time, cycles/ burst and volume were modified, showed no substantial improvement (Table S3, Figure S3).

### Sonication of complex pools of PCR amplicons

**Figure 2:**
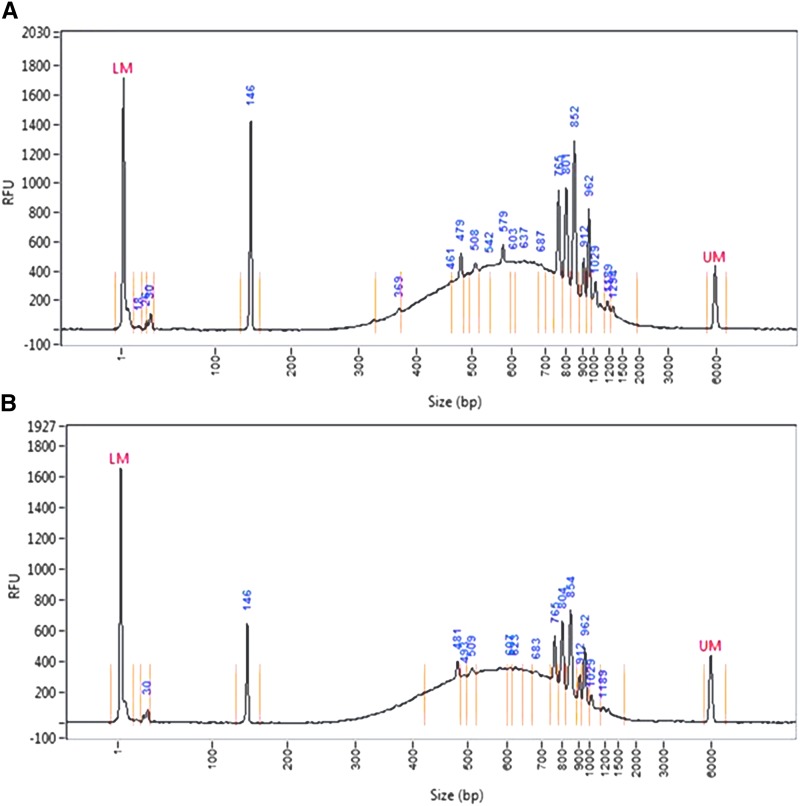
Fragmentation profiles of Illumina sequencing libraries prepared from 32 pooled amplicons produced by PCR amplification of genomic DNA from 256 pooled barley samples. PCR products from forty-eight genomic DNA pools were subjected to sonication using test parameter 13 in Table 1. Fragmentation profiles of two different libraries are shown (top and bottom panel).

Having established optimal conditions for fragmentation of a single amplicon, the parameters were next tested in a TILLING by Sequencing experiment containing 32 amplicons ranging between 670-1513 base pairs. The GC base percentage in the amplicons ranged from 35.16 to 68.66 with a median of 50.28 (Table S1). For comparison, GC values of the entire coding sequence assembly of the barley genome build 2 was calculated as having a minimum GC percentage of 22.86, maximum of 86.96 and median of 52.48 (see methods). The PCR primers producing amplicons tested in this study had been previously validated and used in traditional TILLING assays employing gel-based cleavage assays using an eightfold genomic DNA pooling strategy (Stolarek *et al.* 2015a, 2015b; Szarejko *et al.* 2017). Thus, the amplicon set represents the complexity of a TILLING project, and also allows testing of the feasibility of applying sonication-based fragmentation and next generation sequencing to an already existing TILLING platform. Fragmentation was performed on 48 pools followed by preparation of Illumina libraries (Figure S4). Fragmentation profiles of the prepared libraries showed more complex patterns with average sizes of approximately 650 bp, that includes the size of the target DNA (known as the insert) plus the ligated adapter sequences. The presence of higher concentration peaks at specific molecular weights is also observed (Figure 2).

### Sequencing libraries prepared from pooled fragmented amplicons

**Figure 3:**
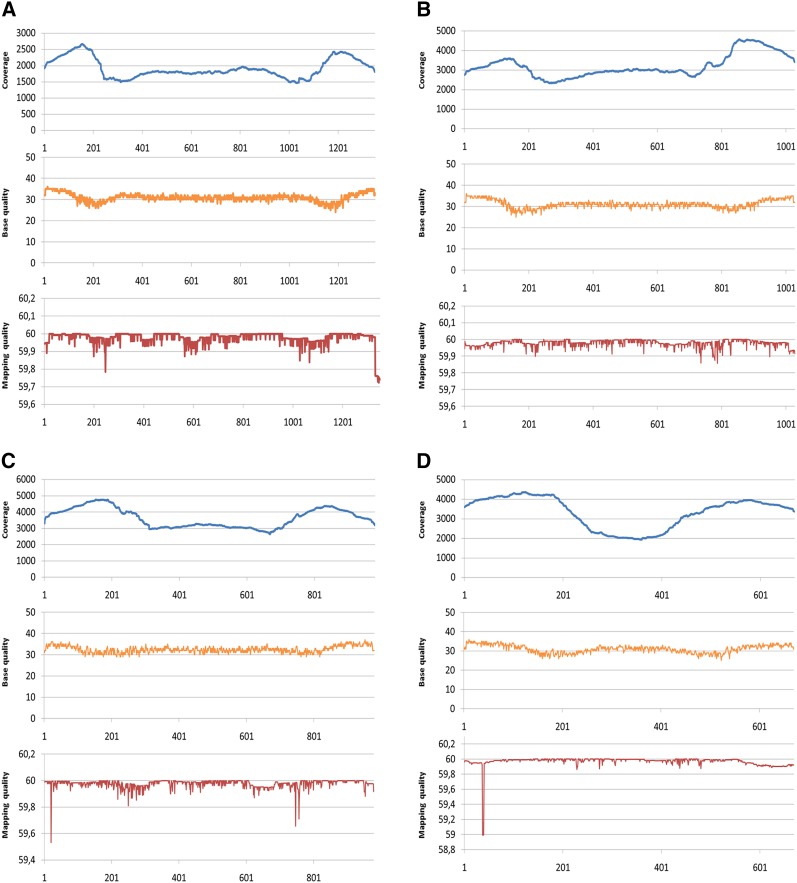
Read coverage, base quality and mapping quality from 2x300PE sequencing of targets EXPB4 (1350 bp) (A), RTH3 (1028 bp) (B), PRT1 (979 bp) (C) and ALS3_1 (670 bp) (D). The number of aligned reads (coverage) at each position in the amplicon is marked in blue. The average base quality per position is marked in yellow and mapping quality in red. Coverage is even across the majority of each amplicon with slight increases in coverage and concomitant decreases in quality score toward the end of each amplicon. Shorter amplicons have longer regions of increased coverage at the ends.

To evaluate the effect of fragmentation on sequencing coverage and quality, the Illumina libraries were subjected to 2x300 PE sequencing on a MiSeq instrument. Sequencing coverage profiles, per base mapping quality, and per base sequence quality of four unique amplicons are shown (Figure 3, Tables S4, S5, S6, S7, S8, S9, S10). Similar data were graphed for two pools and two amplicons from a previously published project where TILLING screening was performed by direct sequencing (without fragmentation) of short amplicons derived from PCR from pools of 64 tomato genomic DNAs (Gupta *et al.* 2017) (Figure S5). The minimum insert size in all 48 barley pools was 259 bp, maximum was 386 bp, and median was 348.23 bp (Table S11). Pools show a distribution of sizes, with accumulation of higher concentration of DNA at specific molecular weights. This is pronounced in the most highly concentrated pool number 24 (Figure S6). At the pool level, mean coverage ranged from 1492 to 50030 with a median of 6365. PCA analysis showed pool 24 to be the outlier with coverage of 50030 (Figure S7). Percent GC ranged from 47.64 to 54.33 and mapping quality from 59.06 to 59.76 (Table S11).

To compare sonicated PCR products to direct sequencing of PCR products, average coverage values for each amplicon in each of the 48 barley pools was tabulated (Table S12). Data were also tabulated from previously published tomato and cassava experiments that employed direct sequencing of PCR products without fragmentation (Table S13, S14) (Duitama *et al.* 2017; Gupta *et al.* 2017). In barley, 1536 coverage values (32 amplicons × 48 pools) were evaluated. The minimum mean coverage was observed in the 1153 bp amplicon named FC1_11 (value = 2093). Maximum coverage was in the 778 bp BAK1_1 (134456), with a mean coverage of 6621 for the total collection of amplicons. A weak negative correlation was observed between amplicon size and mean coverage (*r* = -0.38), and between percentage of GC in the amplicon and mean coverage (*r* = -0.41) (Figures S8, S9 and Table S12). A weak positive correlation was observed between amplicon size and percentage of GC (*r* = 0.25) (Figure S10 and Table S12). To estimate failures, data points were counted where coverage fell below 5% of the mean coverage for the tested amplicon. This detects genomic DNA pools where a single amplicon produces much less coverage than the same amplicon in other DNA pools. Using this criterion, 2.08% of the experiment failed. This is in comparison to 0.5% for tomato (n = 2408) and 1.19% for cassava (n = 2538).

To evaluate the suitability of the experiment to recover induced mutations, the number of times a mutant allele would be sequenced was calculated. In the pooling scheme tested, a heterozygous mutation should be present at a frequency of 0.00195 (1/(256*2)). A coverage of 512 is therefore needed to identify the heterozygous mutation 1 time in a pool. Using this, the percentage of the assay where heterozygous mutations are expected at a specific coverage was calculated. One hundred percent of the experiment produced data for 1x coverage. This reduced to 96.74% for 2x coverage, 88.15% for 4x and 62.30% for 8x coverage (Table S12). Homozygous mutations are twice the concentration in an individual plant and therefore 88.15% of the experiment produced data for recovery of homozygous mutations at 8x coverage.

### Mutation discovery in complex pools

To evaluate the use of sonication for TILLING assays involving complex pooled PCR products, the mapped reads from sequencing were subjected to two mutation discovery pipelines. Three plants harboring previously identified induced mutations were used to evaluate mutation discovery (Szarejko *et al.* 2017). The CAMBa pipeline discovered all positive controls (Table S15). Control 1, a plant harboring a C to T homozygous mutation at position 416 in the PRT1_2 target was identified at allele frequencies of 0.0078 in pool 11 (first dimension of pooling), 0.0103 in pool 19 (second dimension of pooling) and 0.0117 in pool 41 (third dimension of pooling). The expected frequency of a homozygous mutation is 0.0039. Control 2, a plant harboring a C to T heterozygous mutation in target EXPB4_23 was identified in pool 15 (first dimension) at a frequency of 0.00778, in pool 20 (second dimension) at 0.0024 and in pool 37 (third dimension) at 0.00199. The expected frequency of a heterozygous mutation is 0.00195. The heterozygous T to A mutation in control 3, VDE_2_27, was identified in pool 14 (0.0026), 26 (0.0051) and 46 (0.0060). GATK HaplotypeCaller was performed using two different ploidy levels to recover rare alleles. Variant calling at ploidy settings 600 and 2000 both recovered the homozygous mutation in control 1. Allele frequencies in pools 11, 19, and 41 for ploidy 600 were 0.0033, 0.0050 and 0.0067, respectively. For ploidy 2000 the frequencies were 0.0035, 0.0055 and 0.0070. Control 2 was not identified at either ploidy setting. The control 3 mutation was identified only in pool 14 at frequencies of 0.0033 and 0.0030 for ploidy settings 600 and 2000, respectively. No trend was observed between sequencing coverage of the amplicon in the pool and observed *vs.* expected allele frequencies (Table S15).

## Discussion

The ability to rapidly discover novel nucleotide variation in germplasm collections and mutant populations is a fundamental tool for functional genomics and breeding of plants and animals. For example, large germplasm collections have been maintained for many species, but limited resources mean little has been done to characterize gene coding and regulatory sequence variation that may prove important for unlocking their full potential. Further, efficient methods for accurate discovery and cataloguing of nucleotide variation can be important to measure and protect biological diversity in developing countries (Gepts 2004). For functional genomic studies, screening of a TILLING population for the recovery of induced mutations predicted to alter gene function typically requires interrogation of thousands of individuals. While reduced-representation genome methods such as exome capture sequencing have been described to recover rare single nucleotide variants in large genome species such as wheat, the approach remains cost-prohibitive for smaller scale projects and for understudied species where research funds are limited (Krasileva *et al.* 2017). Amplicon sequencing provides a low-cost alternative that is advantageous in that it is highly flexible with regard to population size and the choice of sequence regions for evaluation.

The flexibility and throughput of amplicon sequencing is increased through fragmentation of PCR products. More bases can be interrogated from a PCR product, and fragmentation into small pieces allows use of a range of different short-read sequencing platforms. Fragmentation of low molecular weight PCR products, however, is potentially more challenging when compared to working with genomic DNA where starting fragment sizes can exceed 20,000 bp when using standard genomic DNA extraction protocols (Huynh *et al.* 2017). For example, recovery of a 500 bp fragment from a 1000 bp PCR product requires that a single double strand break be induced at the mid-point of the amplicon. Owing to this, optimal conditions for sonication-based fragmentation of a single amplicon were evaluated. Trials showed that sonication for 30 sec with a peak power of 50 W, Duty factor of 40 and 200 cycles per burst produced a smooth distribution of fragment sizes that peaked near 400 base pairs, a suitable size for Illumina library preparation and sequencing. While different sized amplicons may require subtle optimizations, it was reasoned that sonication at this setting may be suitable for a complex pool of PCR products of varying sizes. A pool of amplicons ranging between 670 and 1513 bp was next evaluated because this is the size range of amplicons used in traditional gel-based TILLING approaches that have served as a benchmark for mutation discovery in pools (Till *et al.* 2003, 2004; Talamè *et al.* 2008; Gottwald *et al.* 2009; Bovina *et al.* 2011). Conditions were sought to allow higher throughput screening of existing TILLING populations where gene targets and oligonucleotide primers have been previously validated.

Evaluation of fragment size distribution of the libraries prepared from pooled PCR products showed a broad distribution of fragments, suggesting suitability for sequencing. Analysis of post-sonication fragment sizes after library preparation, and also of insert sizes post-mapping, showed a broad distribution of fragment sizes with median insert size of 348.23 bp, close 350 bp, one of two optimal insert sizes for Illumina library preparation. Over-accumulation of DNA fragments at specific molecular weights was observed. However, this did not appear to affect the experiment as all pools produced high base and mapping quality with the majority of amplicons in pools producing high read coverage. Over-accumulation of specific molecular weights may indicate that some sequence contexts or fragment sizes are less likely to experience sonication-induced cleavage. The GC content of the amplicon may influence this, as a weak negative correlation between GC content and mean coverage was observed. Internal sequences of DNA fragments longer than the sequencing read length will go unsequenced affecting coverage across an amplicon. However, a weak positive correlation between GC content and amplicon size was also observed, and coverage differences may be explained by the method used for amplicon quantification (see below). Further tests are required to determine if there is any dependence on the concentration of input PCR product, as the phenomenon of accumulation of specific molecular weight fragments was most pronounced in the highest concentration pool number 24. It is interesting to note that while read coverage is generally even across fragmented amplicons, there is a consistent pattern of higher coverage and reduced base quality near but not directly at amplicon termini. This pattern is expected in paired end reads if there is a lower probability for fragmentation of DNA sequences near the termini of the PCR amplicon. Importantly, with the exception of the increases near the amplicon termini, read coverage is consistent across the length of amplicons. A weak correlation was observed between fragment size and median sequencing coverage in the experiment. While the sonication procedure cannot be ruled out as a contributing factor, it is also likely that this observation reflects errors in quantification of PCR products prior to pooling. Intercalating DNA binding dyes will produce brighter band images (with a higher pixel density) in larger DNA fragments compared to smaller fragments of the same molar amount. The quantification used for the barley experiment did not correct for amplicon size. Therefore concentrations were over-estimated for larger fragments and therefore these amplicons were over-diluted compared to smaller fragments. While slightly underrepresented, quantity and coverage from the larger amplicons was suitable for mutation discovery. Based on the consistency of coverage across the length of amplicons, it was concluded that the mean coverage per amplicon in each pool is a useful metric to evaluate the performance of the entire population. The population consisted of 1536 data points (32 amplicons × 48 pools). In total, 2.08% of data points had coverage values less than 5% of the mean for the amplicon in all pools. These were considered assay failures even though some high quality reads were produced. Assay failures measured in this way were slightly lower in cassava and tomato non-fragmented amplicon experiments performed on the same MiSeq sequencer (0.5 and 1.19%, respectively). Assay failures may arise due to a combination of variables including genomic DNA quality, genomic DNA quantification, PCR quality, PCR quantification and library quantification (see below). Interestingly, PCR product evaluation and quantification methods were different in all three experiments. Every tomato PCR product was quantified by capillary electrophoresis using the Fragment Analyzer. This proved costly, and so for the cassava experiment all samples were analyzed using 96 well e-gels and digital image quantification using Image J (Duitama *et al.* 2017; Huynh *et al.* 2017). For barley, the procedure used in cassava was streamlined by replacing digital image quantification with a rapid visual estimation to determine average yield in nanograms for each amplicon. The method of PCR product quantification trends with failure rates based on sequencing coverage, and therefore may be related. Nevertheless, assay failure is low in all experiments and from this we conclude that using a fragmented PCR approach does not significantly add to assay failures.

Comparisons between sequencing metrics from fragmented and non-fragmented PCR products allow further evaluation of the sonication-based approach. Data from direct sequencing of non-fragmented PCR products pooled in a similar fashion (Figure S5) shows both coverage and base quality drop toward the center of amplicons that are near 600 base pairs. In contrast, coverage increases in the center of shorter amplicons as expected when applying paired-end sequencing. Based on this data, we conclude that the sonication-based approach produces more consistent data independent of starting amplicon size. This allows for more precise experimental design, taking into consideration genomic DNA sample pooling and amplicon pooling so that required depth of coverage can be obtained for accurate variant calling.

There are many parameters in addition to amplicon fragmentation that are important for a successful assay. These include proper quantification and pooling of genomic DNA, proper quantification and pooling of PCR products, proper quantification and pooling of sequencing libraries, and producing sufficient read coverage to ensure recovery of rare mutations based on how many genomic DNA samples are pooled together. It is difficult to evaluate if a DNA quality issue results in a subset of genomic DNAs in a pool underperforming in PCR. All other parameters can be investigated. Quantification of PCR products is evaluated through coverage variations within the same amplicon between different pools, and also through coverage variations for all pools combined calculated between different amplicons. Ideally, all PCR products in all pools will be represented at the same molarity and thus produce the same sequencing coverage. This may be impossible to achieve. In the barley experiment, maximum coverage within the same amplicon ranged between 2 and 11 fold higher than the mean (Table S12). Coverage between different amplicons ranged between 2092.7 and 13445.8 (6.42 fold). This is in comparison to a 4.94 fold difference between the minimum and maximum coverage between amplicons in the tomato TILLING experiment. Thus, the careful quantification of each PCR product used for tomato TILLING, which utilized robotic liquid handling and capillary analysis of each non-pooled PCR product, may provide only incremental assay improvements. In addition to PCR concentration, variation in sequencing library quantification will affect the mean coverage of each pool in an experiment. Library quantification in the barley experiment was performed using fluorimetry. One noticeable error was observed whereby pool 24 was highly over-represented (mean coverage of 52170.41) compared to the next highest pool (mean coverage 8854.62, pool 31). Library quantification using fluorimetry allowed rapid quantification compared to quantitative PCR and the over-representation of pool 24 is assumed to have resulted from a human error. Further tests are required to determine if library to library mean coverage can be made more similar by adding additional quantification methods such as qPCR. Nevertheless, assay success can be measured as having achieved sufficient quality and coverage for pools and amplicons in order to recover mutations. Expected variations can be built into the experimental design when choosing the number of PCR amplicons to sequence and the level of genomic DNA pooling to employ. With regards to coverage of induced mutations, 88% of the experiment produced sufficient data for recovery of heterozygous mutations with a read coverage of the mutant allele of at least four. This coverage can be increased by increasing the number of sequencing reads in the experiment.

Numerous DNA sequencers can potentially be used for pooled amplicon experiments when amplicons are fragmented. Quality and throughput can vary between different platforms and between different sequencer models of the same platform, as reported for Illumina sequencers *(*Liu *et al.* 2012). In theory, any platform is suitable provided sufficient coverage and quality is achieved. MiSeq 2x300PE sequencing was used in this study to provide direct comparison with previous non-fragmented amplicon experiments run on the same machine. Higher throughput and lower per-base costs are possible on other sequencing platforms such as the Illumina HiSeq system. Cost estimations can also be considered to further evaluate the utility of sonication of larger amplicons *vs.* direct sequencing. For simplicity, quality and coverage are assumed to be identical for a non-fragmented 600 bp amplicon *vs.* a fragmented 1200 bp amplicon. When considering major consumables and assays employing capillary quantification of all PCR products, it is estimated that it will cost approximately 1.9x more to screen two 600 bp amplicons *vs.* one 1200 bp amplicon that has been sonicated (Table S16). Cost estimations do not take into account the extra time for PCR amplification and FA machine run times when performing more PCR reactions producing shorter amplicon sizes. Comparison of barley, tomato and cassava data suggests that capillary quantification may only provide small assay improvements. Without this step, sequencing of 600 bp amplicons using 2x300PE sequencing is 1.3x more costly than sequencing 1200 bp amplicons (Table S16). Additional time and cost savings may be achieved by using amplicons larger than those reported here. For example, while gene sizes vary, median gene length in plants such as barley can be thousands of base pairs (Wang *et al.* 2017; Mascher *et al.* 2017). An elegant experimental design would be for one amplicon to cover an entire coding sequence. Long PCR, however, can be more challenging to optimize, and careful testing should be performed to avoid unforeseen biases and to ensure that PCR amplification produces and equal representation of molecules from complex pooled genomic DNAs. Errors from PCR must also be considered. Previous studies using traditional gel-based approaches showed using Taq polymerase with amplicon sizes up to 1500 bp resulted in less than a 5% false positive error rate (Till *et al.* 2006a, 2010). PCR based errors are further mitigated when applying a three-dimensional genomic DNA pooling strategy whereby each genomic DNA is represented in three unique pools and true mutations are found only once in each pooling dimension (Tsai *et al.* 2011). When applying this criterion to mutation calling the likelihood of false positive errors due to PCR amplification of genomic DNA or through post-ligation library amplification is reduced. Further, library preparation kits that do not employ post-ligation amplification are available if errors from library preparation are a concern. However, more studies are needed before direct comparisons between traditional enzymatic-based TILLING assays and sequencing-based assays can be made. Higher pooling in sequencing assays (256x *vs.* 8x for enzymatic assays) means that rare errors will be more likely to be detected.

An optimal experimental design for sequencing of fragmented pooled amplicons will balance assay throughput, cost, and accuracy. The ability to maximize recovery of true positives while minimizing false positives is important. The choice of bioinformatics tools is a critical component. Previous studies in tomato TILLING by Sequencing tested six different mutation calling tools and estimated a range of accuracy from 89.33 to 29.33 (Gupta *et al.* 2017). In that study, the CAMBa tool that was purpose built for TILLING performed the best. In the present study, CAMBa successfully identified all three positive controls, but only the homozygous mutation was identified in all three pools using GATK HaplotypeCaller with sample ploidy set at either 600 or 2000. The homozygous mutation is present in DNA pools at an expected frequency of 0.0039 while the heterozygous mutation is expected at 0.00195. Using the amplicon coverage values for the pools, the homozygous mutation is expected to be sequenced 14x in pool 11, 35.65x in pool 19 and 34.26x in pool 41. The heterozygous mutations are expected at 4.98x, 7.91x, 14.26x, 11.81x, 8.04x, and 20.15x respectively, in pool numbers 15, 20, 37, 14, 26, and 46. Lower representation of heterozygous mutations may have contributed to the failure to call the mutation with HaplotypeCaller. Analysis of novel putative mutations identified in the mutant screen is ongoing. True mutations resulting from this work will be used to further evaluate the performance of mutation discovery software. Variant calling tools are constantly evolving and their improvement will likely lead to improvements in pooled amplicon assays such as TILLING.

We conclude that fragmentation of pooled amplicons by ultrasonication provides a suitable method for producing even coverage sequencing for targeted recovery of nucleotide variation in large populations of samples. Alternative methods for amplicon fragmentation, such as use of dsDNA fragmentase have been applied for TILLING with genomic pooling of 64-fold (Burkart-Waco *et al.* 2017). This suggests that multiple methods may be considered for experiments employing complex pools of PCR products. In theory, any method that can produce a distribution of fragments with optimal size ranges for the chosen library preparation method should be suitable. Further tests are needed, however, to determine if alternative fragmentation methods will produce similar results in highly pooled samples. Given all of the experimental variables in a pooled amplicon experiment that can affect variant discovery, and the fact that variant calling algorithms are still evolving, careful testing of amplicon fragmentation, amplicon pooling, genomic DNA pooling and bioinformatics tools using positive controls is advised prior to choosing an experimental design. In addition to recovery of induced mutations it is envisioned that combining sample pooling with amplicon fragmentation can improve screening efficiencies for natural allelic variations. For example, the same approach used for barley mutants can be applied for sequencing of germplasm banks where rare alleles may provide an important resource for understanding gene function and allow for the genetic improvement of domesticated and semi-domesticated species.
